# Use of 3D-DXA in the assessment of bone structure among patients with chronic kidney disease

**DOI:** 10.3389/fmed.2025.1471418

**Published:** 2025-02-04

**Authors:** Martin Kužma, Zuzana Kužmová, Ludovic Humbert, Mirella Lopez Picazo, Roman Králik, Jakub Falat, Juraj Smaha, Peter Jackuliak, Zdenko Killinger, Juraj Payer

**Affiliations:** ^1^5th Department of Internal Medicine, Comenius University Faculty of Medicine, University Hospital Bratislava, Bratislava, Slovakia; ^2^3D-Shaper Medical, Barcelona, Spain

**Keywords:** chronic kidney disease, bone quality, 3D-densitometry, cortical bone, bone mineral density

## Abstract

**Introduction:**

Patients in later stages of chronic kidney disease (CKD) have a 2- to 14-fold increase in fracture risk. Bone mineral density (BMD) assessment is limited due to the inability to measure trabecular and cortical bone characteristics and the interference of aortic calcifications.

**Study objective:**

This study aimed to assess the trabecular bone score (TBS) and three-dimensional dual-energy X-ray absorptiometry (3D-DXA) in participants across all CKD stages.

**Patients and methods:**

In total, 64 CKD patients (consisting of 28 female participants and 36 male participants, with an average age of 69.5 years) were included. There were 9, 12, 8, 9, 11, and 15 participants in stages G1, G2, G3a, G3b, G4, and G5 of CKD, respectively. BMD at the lumbar spine (LS) and proximal femur, as well as the LS TBS, were analyzed. The proximal femur parameters such as cortical and trabecular volumetric (v)BMD, cortical thickness (CTh), and surface (s)BMD at the total hip (TH) and femoral neck (FN) were analyzed using 3D-Shaper software.

**Results:**

Comparison between the earlier stages (G1-G3a) and the later CKD stages (G3b-G5) showed significant differences in carboxy terminal collagen crosslinks (CTx) (386 vs.1053 ng/L), TH areal bone mineral density (aBMD; 0.991 vs. 0.859 g/cm^2^), cortical TH vBMD (831 vs. 795 mg/cm^3^), FN (837 vs. 788 mg/cm^3^), TH cortical sBMD (170 mg/cm^2^), and TH Cth (2.03 vs. 1.92 mm; all *p* < 0.05). Cross-sectional comparisons between each CKD stage showed a gradual decrease in the LS BMD, TH cortical vBMD, sBMD (FN and TH), and TH Cth. Strong positive associations between the glomerular filtration rate (GFR) and cortical parameters (FN/TH vBMD and TH Cth) were observed (*p* < 0.01).

**Conclusion:**

In conclusion, advanced stages of CKD (G3b–G5) were associated with lower cortical bone parameters. The majority of the cortical parameters were correlated with the GFR, demonstrating a direct relationship between the kidney function and bone structure.

## Introduction

Chronic kidney disease (CKD) has been observed to have an increasing prevalence in recent years. One of the important CKD complications is fragility fractures, with a 2- to 14-fold increase in fracture risk depending on the CKD stage. Fractures in individuals with CKD are associated with increased morbidity and mortality (1). Although it is well established that end-stage renal disease (ESRD) is associated with an increased fracture risk (2, 3), earlier CKD stages also pose an increased risk of fractures (4, 5). The gold standard to measure bone mass and predict fracture risk in the general population is dual-energy X-ray absorptiometry (DXA). Some studies have shown that low areal bone mineral density (aBMD) is useful in predicting fractures in CKD stages 3–5 and after kidney transplantation (6–8). However, fractures cannot always be explained by decreased aBMD; they may also be caused by insufficient bone quality. Bone quality is defined by properties of bone material, including bone turnover, microarchitecture, mineralization, accumulation of microdamage, and collagen properties (9). In CKD, these changes in bone quality are called renal osteodystrophy. Several methods have been developed to assess bone quality. In CKD, methods such as high-resolution peripheral quantitative computed tomography (HR-pQCT) were used to assess three-dimensional bone density and structural changes. Additionally, tetracycline double-labeled trans-iliac crest bone biopsy and histomorphometry were peformed to quantify the volume and microarchitecture of cancellous and cortical compartments, microdamage, characteristics of mineralization, turnover, and collagen structure. In patients with CKD, it has been suggested that both aBMD and bone texture, as assessed using the trabecular bone score (TBS), should be evaluated to better estimate the risk of fractures (10). The trabecular bone score, an indirect measure of trabecular bone texture, is associated with a lower estimated glomerular filtration rate (eGFR). Patients with end-stage renal disease (ESRD) had lower TBS compared to healthy controls (11, 12) and these scores were predictive of prior vertebral fractures (13). In individuals with subsequent CKD, a typical finding using HR-pQCT revealed a decrease in cortical volumetric BMD (vBMD) but not in the trabecular bone. This decrease in cortical vBMD is primarily attributable to high levels of parathyroid hormone (14–17). A potentially practical tool for determining cortical and trabecular bone compartments is 3D modeling of the proximal femur DXA scan known as 3D-DXA (18). The accuracy of 3D-DXA structural parameters was evaluated by comparing them with QCT measurements. The results showed strong correlations, with correlation coefficients ranging from 0.86 to 0.96, between the geometric and volumetric structural parameters obtained from 3D-DXA and QCT (18–20). Several studies have revealed the usefulness of 3D-DXA in secondary osteoporosis conditions, including acromegaly (21), rheumatoid arthritis (22), Down syndrome (23), and psoriatic disease (24). In the context of bone changes in CKD, 3D-DXA revealed an impairment of the cortical bone in individuals with primary hyperparathyroidism (25, 26). Recently, in postmenopausal osteoporosis, significant improvements in 3D-DXA measurements, such as cortical thickness, cortical and trabecular volumetric BMD, and surface BMD, were observed in individuals treated with romosozumab followed by denosumab or alendronate (27). A study on denosumab treatment over 5 years in patients with dialyzed CKD (28) revealed lower integral and cortical vBMD values at baseline compared to year 1, with a predominant effect of denosumab on cortical parameters.

In light of these results, we performed a study to evaluate several non-invasive DXA-based methods used for the assessment of trabecular and cortical bone parameters, such as the trabecular bone score (TBS) and 3D-DXA parameters, in individuals across all CKD stages.

## Patients and methods

### Study participants

A single-center cross-sectional study was conducted at University Hospital Bratislava, involving Caucasian participants with chronic kidney disease (CKD) between July 2018 and July 2019. The study protocol was reviewed and approved by the local ethics committee, and informed consent was obtained from all participants prior to their inclusion in the research.

The study population consisted of participants diagnosed with CKD in accordance with the Kidney Disease: Improving Global Outcomes (KDIGO) guidelines, with no restrictions on sex, age, or the underlying etiology of CKD. According to the following KDIGO classifications, participants were categorized based on the estimated glomerular filtration rate (eGFR) as follows: G1 (≥90 ml/min/1.73 m^2^), G2 (60–89 ml/min/1.73 m^2^), G3a (45–59 ml/min/1.73 m^2^), G3b (30–44 ml/min/1.73 m^2^), G4 (15–29 ml/min/1.73 m^2^), and G5 (<15 ml/min/1.73 m^2^). To maintain the focus of the study, individuals with a history of specific osteoporosis treatments, apart from routine vitamin D or calcium supplementation, were excluded.

Additional exclusion criteria were applied to ensure the homogeneity and reliability of the data. These criteria included the presence of acute illnesses, such as infections, or acute exacerbations of chronic diseases, including CKD. In the case of CKD, an exacerbation is defined as an increase in serum creatinine levels exceeding 1.5 times the baseline value from prior examinations. Hospitalized and immobile individuals were also excluded from the study, along with those diagnosed with secondary osteoporosis caused by conditions such as glucocorticoid-induced osteoporosis, malabsorption syndromes, primary hyperparathyroidism, or rheumatoid arthritis.

This carefully selected cohort aimed to provide a clear and controlled analysis of CKD-related factors while minimizing potential confounding variables associated with osteoporosis and acute or secondary illnesses.

### Methods

In all participants, laboratory parameters used to evaluate the stages of CKD (estimated glomerular filtration rate (eGFR) and the albumin/creatinine ratio from a single urine sample), calcium-phosphate metabolism (Ca, P, PTH), additional bone turnover parameters (25-OH-D3, CTx-C-terminal telopeptide fragment of collagen type I), and common biochemical parameters from serum samples were all assessed using electrochemiluminescence immunoassay (ECLIA) with Roche Elecsys 1010/2010 to evaluate the internal environment.

### Bone measurements

Areal (a) BMD measurements in g/cm^2^ at the L1-4 spine (LS), femoral neck (FN), and total hip (TH) were performed using a Hologic Inc., Marlborough, MA, USA. The TBS was obtained from the DXA lumbar spine scans using TBS iNsight® (Medimaps SASU, Pessac, France), version 3.0.2.0. The vertebrae with more than one standard deviation (SD) difference in the BMD T-score compared to the adjacent vertebrae, fractured vertebrae, unreadable LS images, and vertebrae that underwent cementoplasty were excluded from the DXA and TBS analysis. 3D-Shaper-Research (version 2.12.1, 3D-Shaper Medical, Barcelona, Spain) was used to perform a 3D analysis (3D-DXA) from the hip DXA scans. The hip DXA scans were sent to 3D-Shaper Medical, where the analysis was performed by a specialist who was blinded to the CKD grade. 3D-Shaper software was used to assess the trabecular and cortical compartments of the proximal femur, represented by the volumetric (v) BMD in mg/cm^3^, cortical surface (s) BMD in mg/cm^2^, and cortical thickness (Cth) in mm. This 3D statistical model is aligned with the hip DXA scan to create a 3D QCT-like, patient-specific model of the proximal femur. The cortex is subsequently segmented by fitting a function to the density profile computed along the normal vector at each node of the proximal femur surface mesh (29). This fitting function depends on cortical thickness (Cth, in mm), cortical volumetric BMD (cortical vBMD, in mg/cm^3^), the location of the cortex, the density of surrounding tissues, and the imaging blur. Once the cortex is segmented, the trabecular bone part is extracted and characterized as its volumetric BMD (Trabecular vBMD, g/cm^3^). The cortical compartment is characterized by its thickness (Cth) and its volumetric BMD (Cortical vBMD). These two parameters can vary either in the same direction or in the opposite direction (e.g., with teriparatide treatment). To simplify the characterization of the cortical compartment, the cortical surface BMD (cortical sBMD, in mg/cm^2^) is calculated at each vertex of the femoral surface mesh. This is done by multiplying the cortical thickness (Cth in mm) by the cortical vBMD (in g/cm^3^) measured along this thickness. Although a novel approach in the DXA field, cortical sBMD has been previously introduced and used to characterize the cortical compartment of the proximal femur in QCT acquisitions (18, 27, 30–32).

### Statistical methods

For statistical analysis, Analyze-it® version 6.15.4 (The Tannery, Leeds, United Kingdom) was used. Continuous variables were expressed as mean ± standard deviation (SD). Before the statistical analysis, values greater than or lesser than +/− 3 SD were excluded. The Shapiro–Francia test was used to assess the normality of the distribution of the monitored parameters. For individual comparisons between the groups, Student’s (independent sample) t-test and Mann–Whitney U test were performed, depending on the normality of the distribution of the specific data. ANCOVA was used to compare the bone measurements (DXA, TBS, and 3D-DXA parameters) between the individual groups. Correlation analysis was performed to determine the associations between the GFR and bone measurements.

## Results

A total of 64 CKD participants (28 female individuals and 36 male individuals), with a mean age of 69.5 years, were included in the analysis. The participants were grouped across the six CKD stages based on the glomerular filtration rate (eGFR), with 9, 12, 8, 9, 11, and 15 participants in stages G1, G2, G3a, G3b, G4, and G5, respectively. To facilitate comparison, the cohort was divided into two groups: earlier CKD stages (G1–G3a) and later stages (G3b–G5).

Significant differences were observed between the two groups. The participants in the earlier CKD stages exhibited lower levels of creatinine, blood urea nitrogen, serum phosphorus, and CTx, as well as a higher GFR, areal bone mineral density (aBMD), cortical surface and volumetric bone mineral density (vBMD), and cortical thickness at the total hip region, compared to those in the later stages (all *p* < 0.05). However, no differences were observed in age, sex distribution, weight, BMI, albumin-to-creatinine ratio, serum calcium, or other bone measures (Table 1).

**Table 1:** Study group characteristics.

			CKD G1-G3a (*N* = 29)	CKD G3b-G5 (*N* = 35)	*p*-value
	Age (years)		65.9 ± 11.1	66.1 ± 13.9	0.919
	Male/female (*n*)		17/12	19/16	0.203
	BMI (kg/cm^2^)		29.8 ± 4.7	28.2 ± 5.6	0.183
	Weight (kg)		87 ± 15.9	82 ± 20.5	0.188
	eGFR (ml/s)		1.22 ± 0.33	0.382 ± 0.22	<0.001
	Creatinine (μgmol/L)		85.44 ± 17.8	187.2 ± 76.7	<0.001
	Blood urea nitrogen (mmol/L)		6.6 ± 1.7	14.7 ± 7.4	<0.001
	Urine Albumin: creatinine ratio		10.1 ± 47	38 ± 106	0.145
	CTx (ng/L)		386 ± 220	1,053 ± 1,070	<0.001
	Serum calcium (mmol/L)		2.33 ± 0.13	2.33 ± 0.24	0.974
	Serum phosphorus (mmol/L)		1.06 ± 0.16	1.33 ± 0.5	<0.001
	iPTH (pmol/L)		4.63 ± 2.4	5.90 ± 4.3	0.230
	Vitamin D (μg/ml)		17.3 ± 7.5	22.8 ± 7.5	0.022
2D-DXA	Areal BMD (g/cm^2^)	Lumbar spine	1.064 ± 0.86	1.030 ± 0.23	0.538
		Femoral neck	0.836 ± 0.17	0.781 ± 0.15	0.298
		Total hip	0.991 ± 0.15	0.895 ± 0.2	0.060
TBS	Trabecular Bone Score		1.27 ± 0.11	1.32 ± 0.1	0.087
3D-DXA	Trabecular volumetric BMD (g/cm^3^)	Femoral neck	219 ± 61	214 ± 61	0.758
		Total hip	159 ± 41	146 ± 39	0.215
	Cortical volumetric BMD(g/cm^3^)	Femoral neck	837 ± 87	788 ± 98	0.050
		Total hip	831 ± 49	795 ± 61	0.016
	Cortical surface BMD (g/cm^2^)	Femoral neck	144 ± 30	137 ± 28	0.295
		Total hip	170 ± 25	154 ± 30	0.030
	Cortical thickness (mm)	Femoral neck	1.74 ± 0.22	1.77 ± 0.25	0.668
		Total hip	2.03 ± 0.29	1.92 ± 0.25	0.050

n, number; CKD, chronic kidney disease; BMI, Body Mass Index; CTx, carboxy terminal collagen crosslinks; iPTH, intact parathormone; DXA, dual X-ray absorptiometry.

### Bone measures across the CKD stages

#### Areal bone mineral density

**Figure 1:**
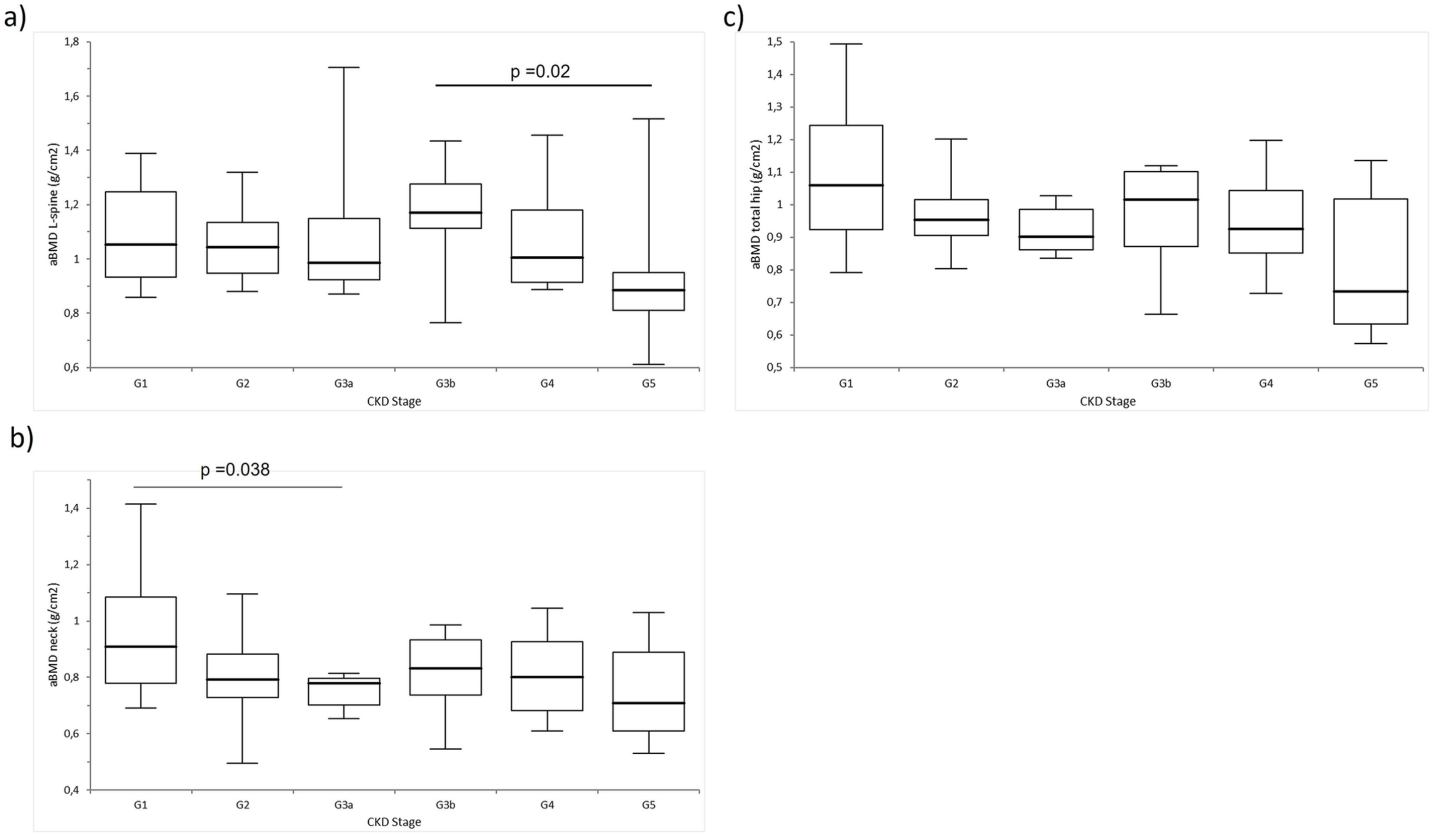
Comparison of the areal bone mineral density (aBMD; g/cm^2^) across the CKD stages G1-G5: **(A)** aBMD at the lumbar spine (L-spine), **(B)** aBMD at the femoral neck (neck), and **(C)** aBMD at the total hip.

Cross-sectional comparisons revealed stage-specific differences in the aBMD. At the lumbar spine, the participants in the CKD G3b stage had significantly lower aBMD compared to those in G5. At the femoral neck, the aBMD was higher in the participants in G1 compared to those in G3a. However, no significant differences in the aBMD at the total hip were identified between the stages (Figure 1).

#### Trabecular compartment

**Figure 2:**
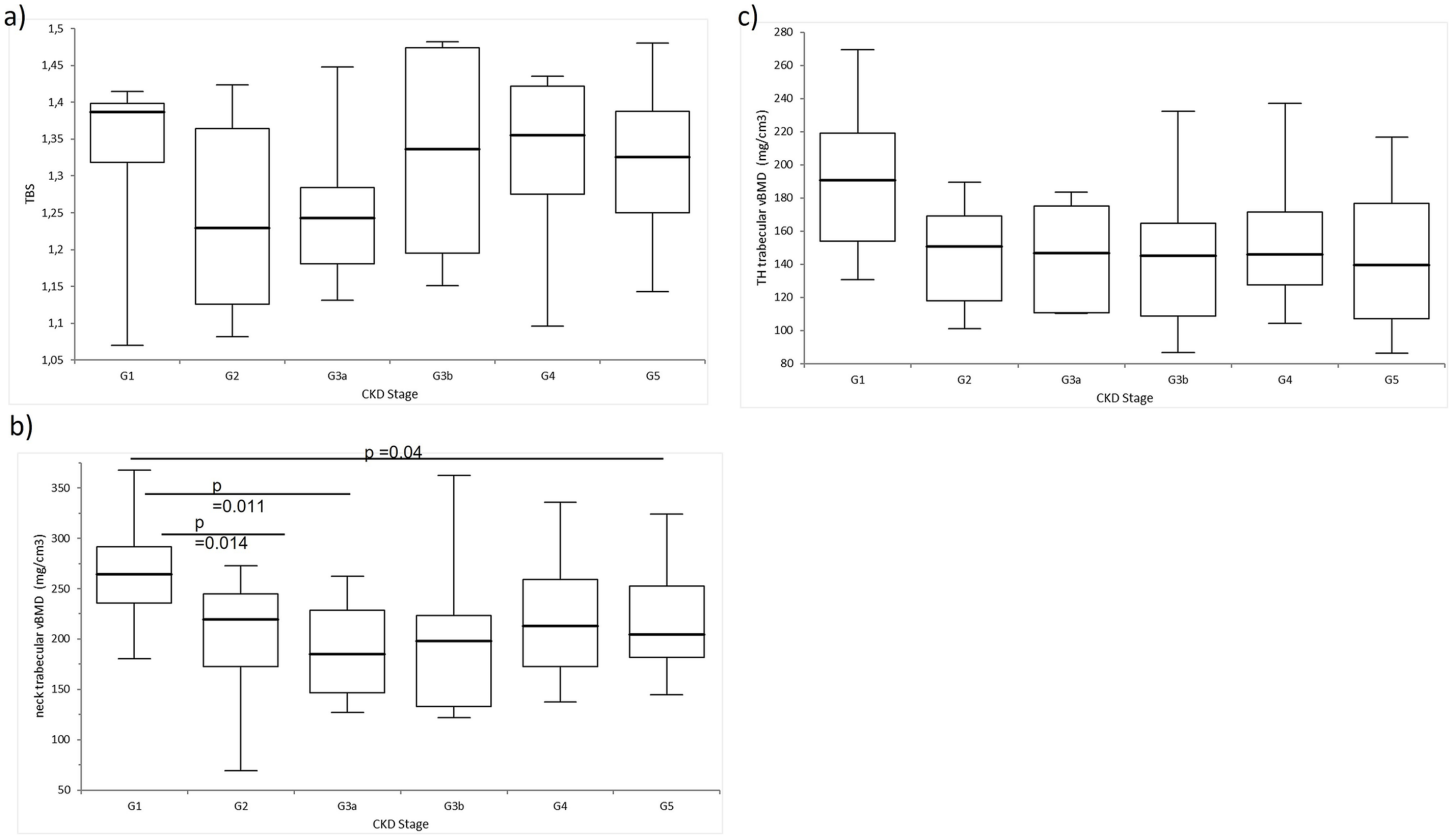
Comparison of the trabecular bone measurements in the study group. There was no difference in the **(A)** TBS and **(C)** trabecular volumetric BMD at the total hip. Significant differences were observed in the **(B)** trabecular volumetric BMD at the femoral neck.

The trabecular volumetric BMD at the femoral neck was significantly higher in the participants in G1 compared to those in G5, G3a, and G2. No significant differences were observed in the trabecular volumetric BMD at the total hip or in the trabecular bone score (TBS) values across the CKD stages (Figure 2).

#### Cortical compartment

**Figure 3:**
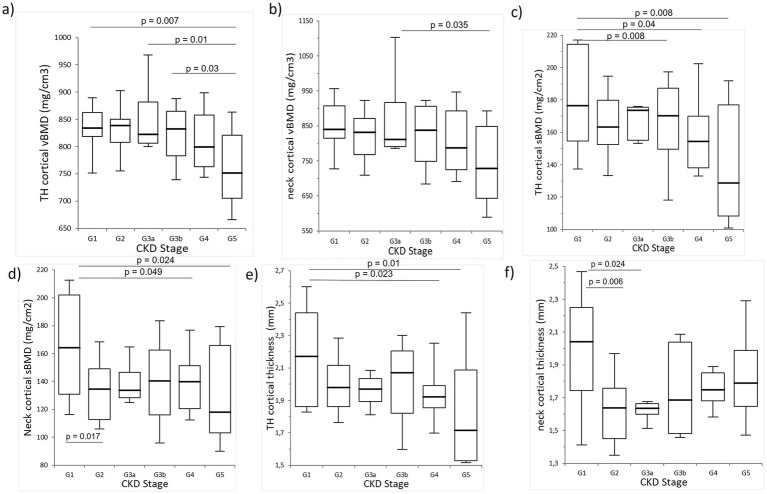
Comparison of the cortical bone measurements. A significant trend was observed with a gradual decrease in the cortical volumetric BMD at **(A)** the total hip and **(B)** the neck, **(C)** cortical surface BMD at the total hip, **(D)** cortical surface BMD at the femoral neck, and cortical thickness at the **(E)** total hip and **(F)** femoral neck.

The cortical measures demonstrated a declining trend with the advancing CKD stage. The cortical volumetric BMD at the total hip was significantly lower in the participants in the G5 stage compared to those in G3b, G3a, and G1 stages. Similarly, the cortical surface BMD at the total hip was significantly greater in the participants in G1 compared to those in G3b, G4, and G5. The cortical surface BMD at the femoral neck and cortical thickness at the total hip were also higher in the participants in G1 compared to those in G2, G4, and G5. In addition, the femoral neck cortical thickness in the participants in G1 was significantly greater than those in G2 and G3a. No other significant differences were observed (Figure 3).

#### Correlations between the GFR and bone measures

**Figure 4:**
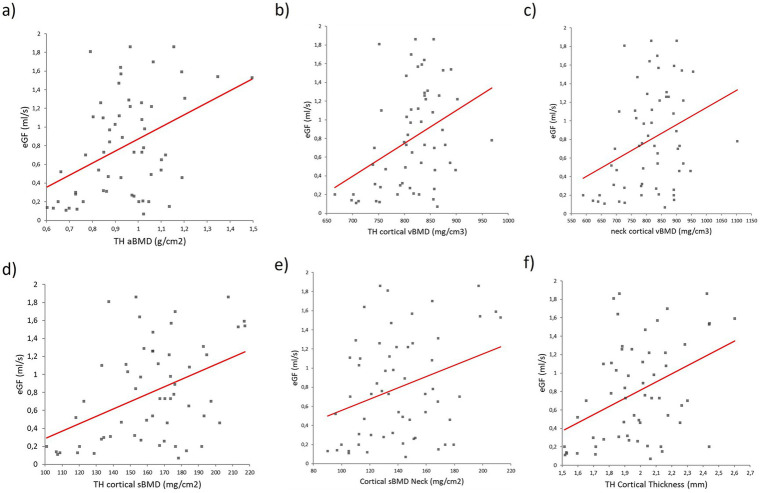
Regression analysis between the eGFR and (a) aBMD at the total hip, **(B)** cortical volumetric BMD at the total hip, **(C)** cortical volumetric BMD at the femoral neck, **(D)** cortical surface BMD at the total hip, **(E)** cortical sBMD at the femoral neck, and **(F)** total hip cortical thickness.

**Figure 5:**
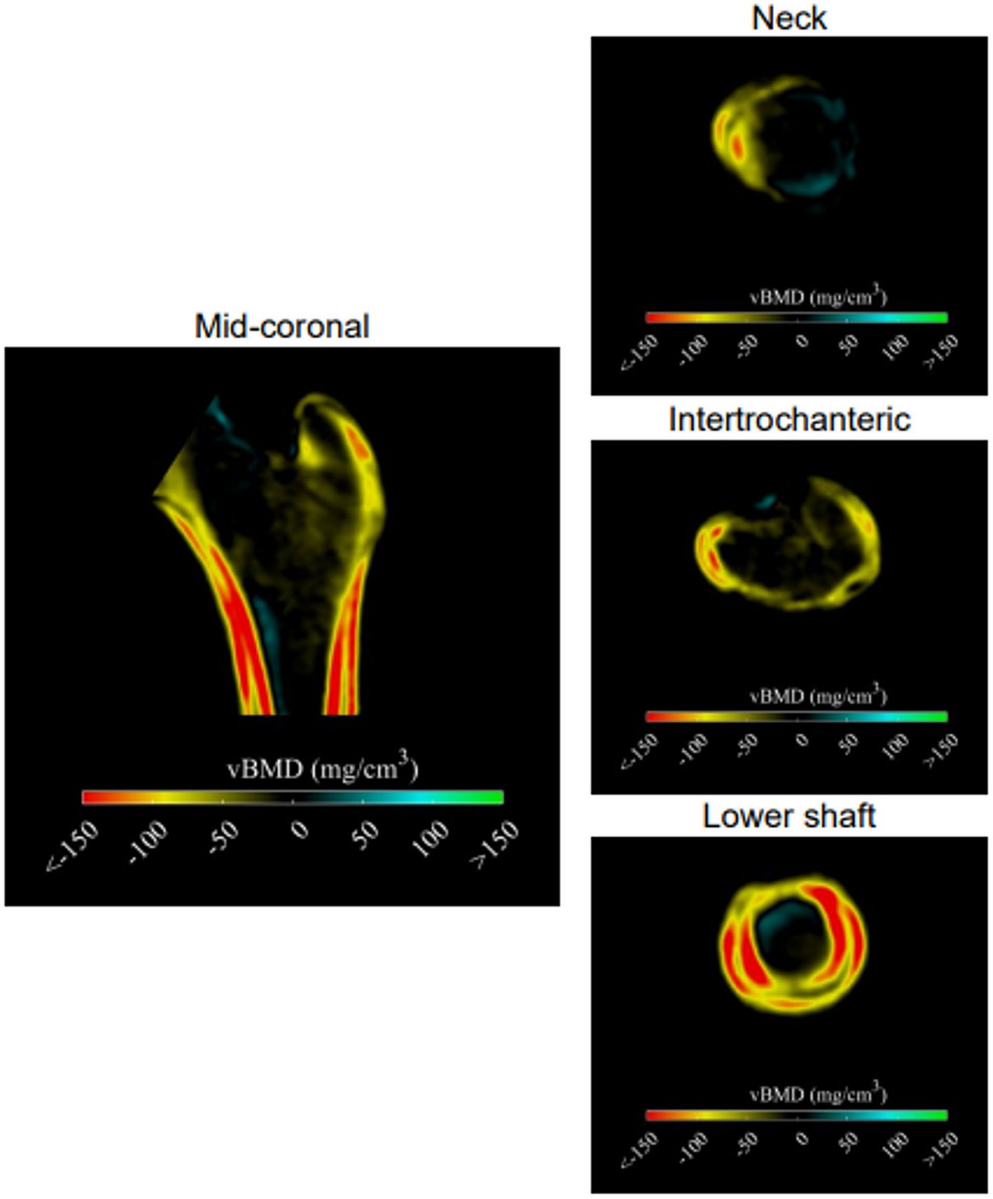
Anatomical distribution of the differences in the bone structure. Differences in the cortical and trabecular vBMD are displayed using cross-sectional images. The higher values in the case group compared to the control are presented in blue and green colors; the lower values in the case group compared to the control are presented in yellow and red colors.

Across all the participants, the eGFR positively correlated with several bone parameters. Notable associations included the aBMD at the total hip (R = 0.42, *p* = 0.001), cortical volumetric BMD at the total hip (R = 0.38, *p* = 0.003) and femoral neck (R = 0.33, *p* = 0.01), cortical surface BMD at the total hip (R = 0.43, *p* = 0.0008) and femoral neck (R = 0.31, *p* = 0.015), and total hip cortical thickness (R = 0.41, *p* = 0.001). In contrast, no correlations were observed between the eGFR and trabecular volumetric BMD or TBS (Figures 4, 5).

## Discussion

Every fragility fracture in patients with CKD leads to decreased quality of life and can even be life-threatening. Therefore, a simple and easy-to-use method for assessing fracture risk in patients with CKD is necessary. The gold standard in the general population is DXA. However, the use of DXA in CKD is ambiguous because it does not provide information about bone tissue quality or the type of renal osteodystrophy. Studies assessing bone quality have shown significant impairment of the cortical compartment, characterized by decreased cortical thickness and increased porosity, in patients with advanced CKD (14–17). In this study, we used DXA-based areal measurements of BMD, TBS, and 3D-DXA measurements to assess the bone structure of the participants with CKD. Lower aBMD, cortical surface BMD, volumetric BMD, and cortical thickness at the total hip were observed in participants in the G3b-G5 CKD stages compared to those in the G1-G3a stages. In addition, markedly decreased cortical parameters, but not trabecular parameters, as measured by 3D-DXA, were observed across all CKD stages. An association between the cortical bone measurements and eGFR was observed.

According to these results, cortical bone measurements by 3D-DXA could potentially be used in clinical practice as an alternative to HR-pQCT or bone biopsy for assessing bone structural changes in CKD patients. Differences in cortical parameters, especially between cortical sBMD and cortical thickness, highlight the potential of the 3D-DXA method to predict femoral neck fractures, which have the highest prevalence in the later stages of CKD (10). In a study comparing bone histomorphometry and micro-CT in patients with CKD G5 and 5D, it was found that cortical porosity and cortical thickness correlated with parathyroid hormone values (33).

In patients with CKD G5 on dialysis, an association between hip aBMD and cortical porosity from bone biopsy was observed (34), possibly due to the high volume of the cortical bone in the proximal hip region. In this study, the TH aBMD was lower in the later (G3b-5) CKD stages and was also associated with the eGFR, thereby supporting the findings of the above-mentioned study. aBMD has limited applicability in characterizing different types of bone disease in CKD-MBD, as all types of bone disease can exhibit a similarly reduced T-score. The interpretation of aBMD may be biased by the presence of extraosseous calcifications, which may overestimate the final values. In addition, DXA measures the density of bone minerals in a specific area and cannot distinguish between the cortical and cancellous bones.

Bone biopsy is an invasive method that requires special equipment and experienced staff, but it is also the most reliable test for diagnosing various forms of renal osteodystrophy (35). Although QCT was observed to correlate with histomorphometrically assessed trabecular bone volume, thickness, number, and separation were observed (36). Micro-CT was found to be less effective in distinguishing between normal bone or renal osteodystrophy (37). Correlations were found between cortical width and cortical thickness, as measured by HR-pQCT and bone biopsy, in participants with hyperparathyroidism or postmenopausal osteoporosis (38). In addition, geometric and volumetric bone measurements, as assessed by QCT, showed a significant correlation with 3D-DXA (20). In a study involving 70 participants with CKD stages 2–4, compared to healthy controls, significant trabecular impairment was observed in both genders, while cortical impairment was observed in men, as assessed by HR-pQCT (39). Pre-dialysis participants with CKD and fractures were reported to have lower aBMD, thinner cortex, and lower trabecular bone volume, as determined by HR-pQCT, compared to non-fractured pre-dialysis CKD participants (40, 41). In our previous study (42), a gradual decrease in the TBS was observed, but only in the G1-G3a stages of CKD. In this study, there was a marked decrease in the cortical vBMD, cortical sBMD, and cortical thickness in the later CKD stages. In addition, a significant correlation between the cortical parameters and eGFR was observed, demonstrating the relationship between the cortical bone and kidney function.

This study has several limitations, including its cross-sectional design, a small number of participants across the CKD stages, and the absence of a control group and fracture data. In addition, the distribution of the sample size among the CKD stages was insufficient. This imbalance might have affected the stability of the statistical results and the generalizability of the conclusions. The cross-sectional design did not allow us to establish causality; it only allowed us to identify associations between the parameters related to bone structure across different CKD stages.

However, we must emphasize that, to the best of our knowledge, this is the first study assessing the use of 3D-DXA modeling across all non-dialysis stages of CKD.

In conclusion, this study shows the use of the DXA-derived method, 3D-DXA, for the indirect assessment of bone microstructure in patients with CKD. Lower aBMD, cortical surface BMD, cortical volumetric BMD, and cortical thickness at the total hip were observed in patients with later CKD stages, compared to those in the earlier stages. Notably, a decreasing trend in the cortical, but not trabecular, parameters, as measured by 3D-DXA, was observed in the advanced CKD stages. In addition, a significant positive correlation between the GFR and cortical parameters was observed, showing an association between the cortical bone and kidney function. It is plausible that 3D-Shaper (former 3D-DXA) could be used in routine practice to better assess the bone quality, thereby contributing to improved fracture risk prediction. However, further longitudinal studies that include fracture assessments are needed.

## Data Availability

The raw data supporting the conclusions of this article will be made available by the authors, without undue reservation.
